# Pulmonary Silicosis Presents with Pleural Effusion

**DOI:** 10.1155/2015/543070

**Published:** 2015-09-07

**Authors:** Mohsin Salih, Tarake Aljarod, Mohamed Ayan, Melnick Jeffrey, Bobby H. Shah

**Affiliations:** ^1^Internal Medicine Department, St. Luke's Hospital, Chesterfield, MO 63017, USA; ^2^Internal Medicine Department, Morehouse School of Medicine, Atlanta, GA 30310, USA; ^3^Internal Medicine, Creighton University Medical Center, Omaha, NE 68102, USA

## Abstract

Silica and silicate mineral dust inhalation can cause a variety of histopathological changes in the lungs and pleura. These include pulmonary silicotic nodules, interstitial infiltrate, fibrosis, and pleural thickening. Pleural effusion is an extremely rare presentation of silicosis. To our best knowledge, there have been only 2 cases of silicosis with pleural effusion reported in medical literature. Herein, we describe a case of a 77-year-old male with almost 50 years' history of occupational silica exposure. He presented with a 4-week history of exertional shortness of breath. He is a lifetime nonsmoker, with no known other significant pulmonary disease. He had chest X-ray which showed a right lung infiltrate and bilateral pleural thickening and effusion. Chest CT showed moderate-sized bilateral pleural effusion and thickening with multiple bilateral intrapulmonary nodules seen. He had undergone extensive workup and was diagnosed with silicosis.

## 1. Introduction

Various pleural involvements such as pleural thickening and progressive multifocal fibrosis (PMF) associated pleural invaginations are well-recognized complications associated with silicosis, particularly advanced pulmonary silicosis. However, pleural effusion is not a well-recognized finding in patients with silicosis. To the best of our knowledge, there have been only 2 cases reported in the medical literature that described pulmonary silicosis presented with pleural effusion. Herein, we describe a case of a 77-year-old gentleman who presented with shortness of breath and bilateral pleural effusion. The patient had undergone extensive workup and was diagnosed with pulmonary silicosis.

## 2. Case Presentation

**Figure 1:**
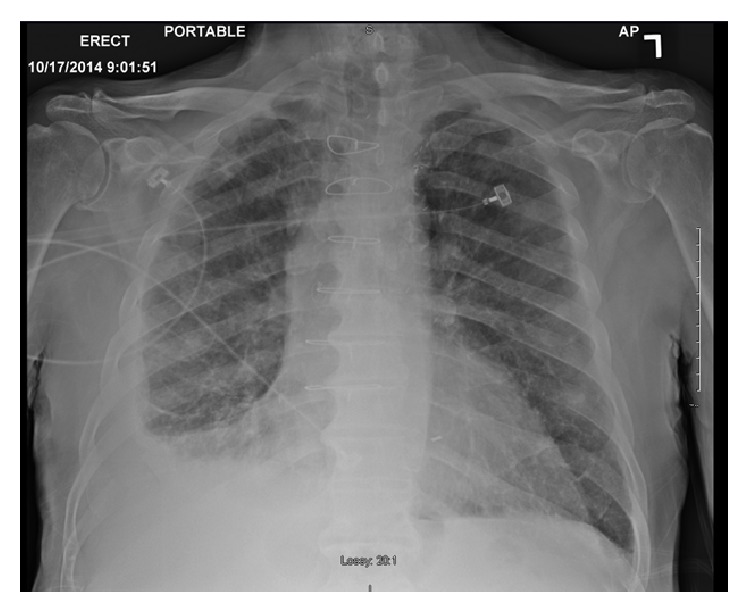
Chest X-ray showed a right lung infiltrate and moderate right pleural effusion, small left pleural effusion, bilateral small irregular opacities, and pleural thickening.

**Figure 2:**
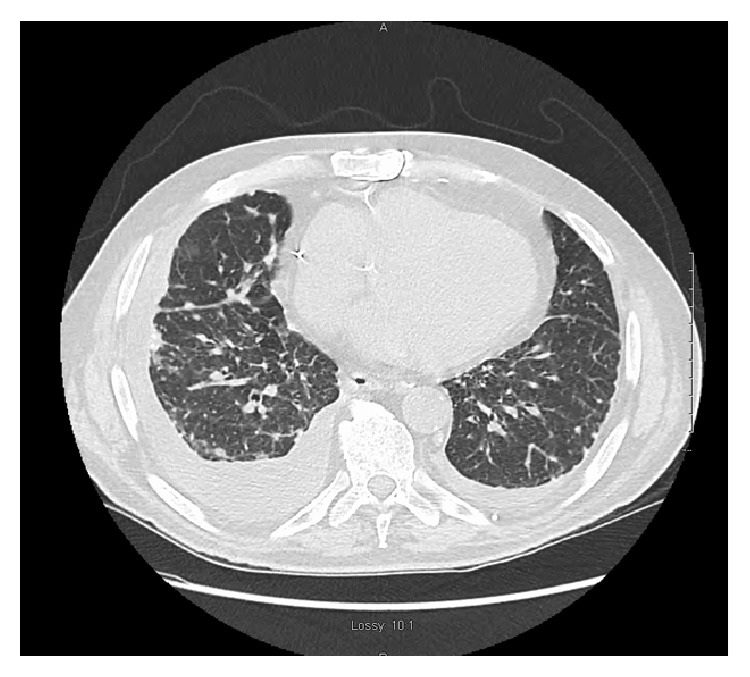
Chest CT showed moderate-sized bilateral pleural effusion and thickening with pulmonary nodules. There is mild interstitial fibrosis. There are multiple bilateral intrapulmonary nodules seen. Some of the nodules are pleural-based. A few nodules do have a small amount of associated calcification.

**Figure 3:**
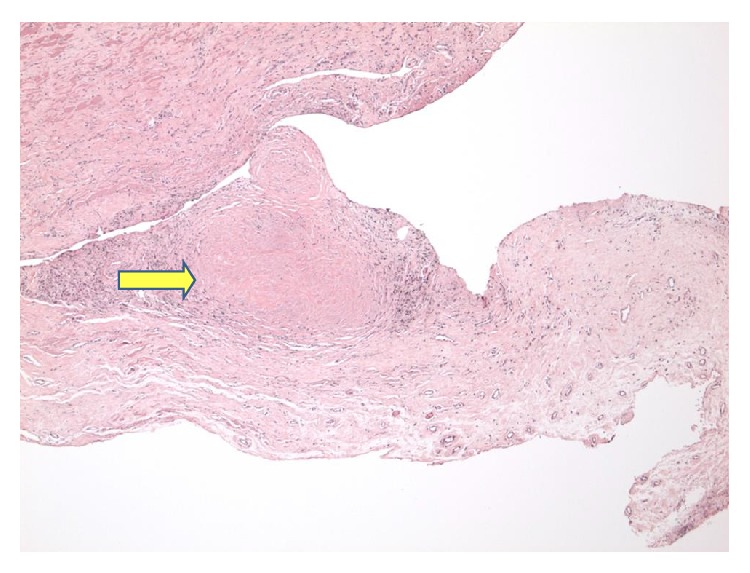
Lung biopsy showed a silicotic nodule within lung parenchyma composed mainly of bundles of interlacing collagen. There is minimal inflammatory reaction.

**Figure 4:**
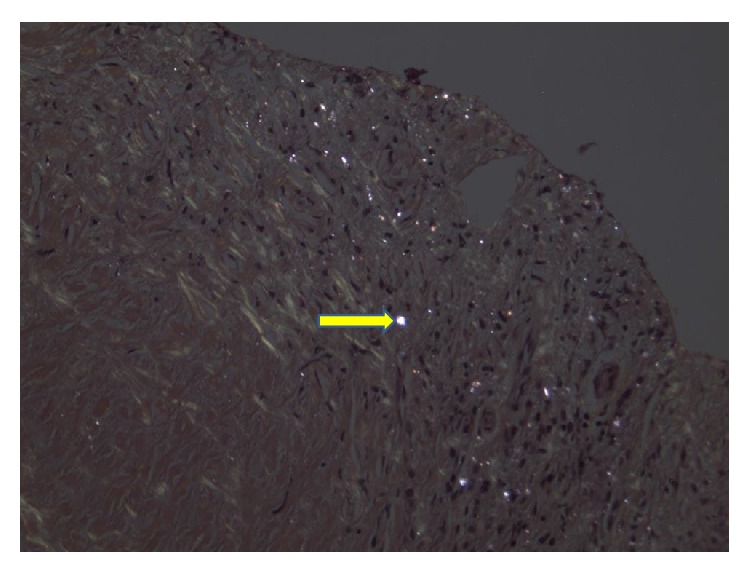
Lung biopsy underpolarized microscopy showing bright white silica crystals of varying sizes.

A 77-year-old gentleman with history of coronary artery disease, diabetes mellitus, and hypertension presented to the emergency department with worsening shortness of breath. He reported that he has been having some chronic baseline dyspnea on exertion which has been stable until 4-5 days prior to his presentation when it started to get worse with simple tasks such as getting dressed and showering. He denied any cough, sputum production, hemoptysis, paroxysmal nocturnal dyspnea, orthopnea, leg swelling, fever, chills, weight loss, or night sweating. He is a lifetime nonsmoker, with no other significant pulmonary history other than his occupational exposure. He had worked for at least 50 to 60 years carving and grinding stones, including granite and numerous other stones. Although he has been wearing a respirator for a large part of his more adult life, there was quite some time, including numerous years, where he wore no respiratory protection whatsoever. He has no tubercular risk factors other than silica exposure. There was no history of TB exposure. His past medical history is notable for coronary artery disease, diabetes, chronic kidney disease, and hypertension. There was no family history of lung cancer. On examination, he was afebrile and tachypneic with respiratory rate of 30. His oxygen saturation was 95% on room air at rest. On chest exam, he had signs of bilateral pleural effusion. Pertinent lab studies showed WBC count of 9 k/*μ*L, creatinine of 2.8 mg/dL, albumin of 3.6 g/dL, total protein of 7.4 g/dL, and BNP of 216, and the arterial blood gas was normal. He had chest X-ray which showed a right lung infiltrate and moderate right pleural effusion, small left pleural effusion, bilateral small irregular opacities, and pleural thickening (Figure 1). Chest CT showed moderate-sized bilateral pleural effusion and thickening with pulmonary nodules. There is mild interstitial fibrosis. Some of the nodules are pleural-based (Figure 2). A few nodules do have a small amount of associated calcification. The largest nodule measures 6 mm. No progressive massive fibrosis (PMF) is defined as the presence of pneumoconiotic nodules more than 2 cm in diameter on CT image. Transthoracic echocardiography showed normal ejection fraction of 60–65% and no significant valvular lesion. He subsequently had a thoracentesis. The pleural fluid was clearly exudative with LDH effusion/LDH serum ratio of 2.15 and pleural fluid LDH of 1205 IU/L. The pleural fluid analysis showed also pH of 7.17, RBC count of 60,000/microL, and WBC count of 871/microL which were mainly lymphocytes. Flow cytometry did not show any evidence of lymphoma. The pleural fluid Gram staining was negative for any bacteria. Histoplasma antigen and histoplasma immunodiffusion were negative. The PDD test was negative. Cultures of the pleural fluid and BAL were unremarkable for any bacteria, TB, or fungi. He had video-assisted lung biopsy, chest tube placement, and mechanical pleurodesis. Lung biopsy (thoracoscopic wedge resection) showed a silicotic nodule within lung parenchyma composed mainly of bundles of interlacing collagen. There is minimal inflammatory reaction (Figure 3). Lung biopsy under polarized microscopy showed bright white silica crystals of varying sizes (Figure 4). Pleural biopsy showed fibrinous exudate with organizing fibrosis and mild acute and chronic inflammation and hyperplasia of mesothelial cells. Pleural biopsy culture for tuberculosis was negative.

## 3. Discussion

Silicosis is an occupational lung disease caused by inhalation of free crystalline silicon dioxide or silica. The pulmonary silicosis is a well-known occupational lung disease caused by silica inhalation; however, new cases of pulmonary silicosis are still seen in clinical practice. Phagocytosis of crystalline silica in the lung causes lysosomal damage, activating the NALP3 inflammasome and triggering the inflammatory cascade with subsequent fibrosis [1].

Occupational silicosis has both pulmonary and extrapulmonary manifestations. Involvement of pleura is well described in pulmonary silicosis. Various pleural involvements can occur especially in advanced pulmonary fibrosis [2, 3]. Extrapulmonary involvement of multiple organs was reported including liver, kidney, spleen, hilar and extrathoracic lymph nodes, and tonsillar and peritoneal involvement. Tuberculosis is a well-established complication of silicosis. Silicosis was also found to be associated with some chronic kidney diseases [4], rheumatoid arthritis, systemic sclerosis, and a human carcinogen [5, 6].

Diagnosis of silicosis generally relies on 3 key elements: a history of substantial exposure to silica dusts with appropriate latency from the time of first exposure, compatible radiological features, and exclusion of other competing diagnoses, such as miliary tuberculosis, fungal infections, sarcoidosis, idiopathic pulmonary fibrosis, other interstitial lung diseases, and primary or secondary lung cancers [1]. Several pulmonary clinical presentations have been described including acute, accelerated, and chronic silicosis. The onset of the presenting symptoms in relation to the first exposure to silica and the radiological features play an important role differentiating the above-mentioned pulmonary involvements.

As mentioned above, pleural involvement in silicosis is well recognized especially in advanced pulmonary silicosis. However, pleural silicosis is still less common and less emphasized in medical literature than asbestosis as an occupational lung disease that is associated with pleural plaque, pleural effusion, and diffuse pleural thickening [7]. The pleural disease in silicosis has not, to our knowledge, been extensively described in the literature as a possible underlying cause for pleural effusion in patients with significant occupational exposure to silica or silicates. After doing a thorough medical literature review, with the exception of 2 case reports and 1 retrospective study that evaluated the extent of pleural diseases in autopsy-proven silicosis, we did not find any review articles or online resources that mentioned pleural effusion as one of the clinical manifestations with which silicosis patients can present (Table 1) [2, 7]. Al-Kassimi reported a case of advanced pulmonary silicosis who was found to have pleural effusion upon presentation. The pleural biopsy was significant for mesothelial hyperplasia [7]. Zeren et al. also reported unusual presentation of silica-associated pleural effusion and pleuritis indicating preferential involvement of the pleura. Like our case, the pleural biopsy reported by Zeren et al. showed pleural thickening and fibrosis. However, it was different from our case and the case reported by Al-Kassimi because there was no lung involvement at both radiological and pathological levels.

Arakawa et al. conducted a retrospective study on the images of 110 patients who had an autopsy-proven silicosis. They found that pleural effusion is present in 35% of the patients studied. Sixty-eight percent (26 of 38) of the patients with pleural effusion had possible/probable cause for their effusion that was not silicosis (pneumonia, cardiac failure, hypoalbuminemia, pneumothorax, and malignancy) and only 11% of the silicosis patients had pleural effusion that is thought to be related to silicosis (no other causes have been found) [8]. In any case of silicosis, based on this case series, the majority of pleural effusions associated with silicosis will be due to a cause other than silicosis. In addition to pleural effusion, Arakawa et al. described some other pleural diseases in silicosis such as pleural thickening and PMF associated pleural invagination. In advanced silicosis, the pleural surface is thickened and is fibrotic with typical silicotic nodules and plaques. However, it was a retrospective review of medical records and so there was no uniform approach to work up the causes of the effusion.

This patient met diagnostic criteria for pulmonary silicosis mentioned above in terms of his job-related prolonged exposure to silica, bilateral pulmonary nodules on both CXR and CT scan, and absence of other alternative conditions such as primary or secondary malignancies, TB, or fungal infections. This patient likely had chronic silicosis given his remote history of silica exposure and his chronic history of dyspnea. Probably, the moderate-sized pleural effusion is what makes his shortness of breath worsen and present in acute pattern as we ruled out all possible infectious causes.

## 4. Conclusion

Different pleural disease can occur in silicosis particularly in advanced stages. Although pleural effusion is unusual in silicosis it is a possible cause if infectious, malignant, cardiovascular, renal, and connective tissue disease causes have been ruled out.

## Figures and Tables

**Table 1:** Summary of the reported cases of silicosis presenting with pleural effusion.

Case	Al-Kassimi's case [7]	Zeren et al.'s case [2]	Our case
Age/sex	70-year-old male	57-year-old male	77-year-old male

Occupation	Well digging, no more exposure	Plumbing fixture factory where he sprayed glazing compound, still exposed	Carving and grinding stones, no more exposure

Occurrence of pleural effusion in relation to time of silica exposure	More than 40 years	32 years	More than 50 years

Onset of symptoms	Insidious	Subacute	Acute on chronic

Presenting symptoms	Shortness of breath	Shortness of breath, pleuritic chest pain, and fever	Shortness of breath

Radiographic changes of patient regarding silicosis	Chest X-ray showed bilateral interstitial shadowing more pronounced in the upper zones with massive pulmonary fibrosis as well as right pleural effusion (simple silicosis)	The chest X-ray and CT scans showed thickening of the left pleura consistent with a chronic process and a small right pleural effusion (simple silicosis)	The chest X-ray and CT scans showed bilateral pleural effusion and thickening with bilateral small irregular opacities (simple silicosis)

Transbronchial biopsy	Birefringent particles compatible with silicosis; hyperplasia of mesothelial cells	Macrophage containing birefringent particles; hyperplasia of mesothelial cells	Histiocytes containing refractile foreign material, consistent with silicosis; hyperplasia of mesothelial cells

Pleural fluid analysis	Exudative pleural effusion with high LDH	Not reported	Exudative pleural effusion with high LDH

Treatment & outcomes	Chemical pleurodesis; no recurrence in 3.5 years	Patient changes his job and his symptoms spontaneously resolved, resolved in 1 month, no recurrence in 3 years	Mechanical pleurodesis; no recurrence in 12 months
